# What They Are Doing in Scotland

**Published:** 1861-05

**Authors:** 


WHAT THEY ARE DOING IN SCOTLAND.—According to a return of the Registrar-General, during the year 1860, 105,704 births were registered in Scotland; 9651 of the births were illegitimate, giving the proportion of 9·1 per cent. of the births as illegitimate, or 1 illegitimate in every 10·7 births.

